# Mental health of diplomatic personnel: scoping review

**DOI:** 10.1093/occmed/kqad032

**Published:** 2023-03-09

**Authors:** S K Brooks, D Patel, N Greenberg

**Affiliations:** Department of Psychological Medicine, King’s College London, Weston Education Centre, London SE5 9RJ, UK; Overseas Health and Welfare, Foreign, Commonwealth and Development Office, King Charles Street, London SW1A 2AH, UK; Department of Psychological Medicine, King’s College London, Weston Education Centre, London SE5 9RJ, UK

## Abstract

**Background:**

Diplomatic personnel frequently relocate as part of their roles, requiring them to adapt to various cultural and political conditions; many are also at risk of experiencing trauma from being deployed to high-threat postings. With diplomatic personnel having to balance the usual pressures of their work with the uncertainties of COVID-19 in recent years, it is particularly important now to understand how to protect their mental health.

**Aims:**

To synthesize existing literature on the well-being of diplomatic personnel to improve understanding of how to protect their mental health.

**Methods:**

A scoping review was carried out to explore what is already known about the well-being of staff working in diplomatic roles. Four databases were searched and reference lists, as well as one key journal, were hand-searched.

**Results:**

Fifteen relevant publications were included. There was little consensus as to how the psychological well-being of diplomatic personnel compares to other populations or which factors predict well-being. Diplomats’ psychological responses to traumatic experiences appeared similar to those of other trauma-exposed occupational groups.

**Conclusions:**

Further research is needed to better understand the well-being of diplomatic personnel, particularly those not deployed to high-threat posts.

## Teaser

This scoping review found little consensus as to how the psychological well-being of diplomatic personnel compares to other populations, which factors predict well-being, or how to best support those deployed to high-threat postings. However, insights from other organizations suggest that diplomatic organizations are likely to benefit from ensuring that all those in supervisory/management positions have the skills and confidence to speak to team members about their psychological well-being.

**Key learning points**

**What is already known about this subject:**

- Frequent international travel for work can have negative psychological consequences for employees.
- The COVID-19 pandemic is likely to have been particularly difficult for employees posted overseas, whose work requires adjustment to the host country and typically involves frequent social events.
- Diplomatic personnel are an example of an occupational group whose work involves frequent international travel and is likely to have been profoundly affected by the pandemic; however, little is known about the well-being of this group.

**What this study adds:**

- Diplomatic personnel are not a well-researched population and there is limited evidence available relating to the prevalence of mental health problems in this occupational group or the factors predictive of their well-being. Despite deployment to high-threat postings being a regular occurrence for some diplomatic staff, there appears to be little consensus across organizations as to how best to support these employees.
- Research on trauma-exposed diplomats suggests their post-traumatic experiences are similar to those of other occupational groups, such as disaster relief workers, and that they experience both post-traumatic stress symptoms and post-traumatic growth.

**What impact this may have on practice or policy:**

- Further research on the mental health of diplomatic personnel in non-hardship posts, the factors associated with diplomats’ mental health, psychosocial interventions for diplomats and the experiences of diplomats during the COVID-19 pandemic is needed.
- In the meantime, diplomatic organizations can draw on insights from other occupational groups in terms of how to support employees during prolonged crises. Research with other occupational groups highlights the importance of managerial support; team cohesion and peer support; and recognition of staff performance. These factors may be particularly helpful for diplomatic organizations to consider in the aftermath of the COVID-19 pandemic.
- Support from within the organization is likely to be a major factor in protecting the mental health of diplomats and enhancing their resilience to work during a prolonged crisis such as COVID-19.

## Introduction

Occupations involving frequent international travel present unique job demands, including potential language barriers; difficult decisions regarding either uprooting families or leaving them behind; disruption to support systems; adaptation and adjustment to different cultures; and missing home, family and friends [1,2]. A recent review concluded that international work can have negative consequences for psychological, physical and physiological health [3].

Diplomatic personnel—staff who work for diplomacy services managing international relations and enabling states to accomplish their foreign policy objectives—are a relatively under-researched population, whose work involves frequently relocating and working in countries with various social, economic, political and cultural conditions. Many are deployed to high-threat postings which can expose them to various potentially traumatic situations, including being targets of violent attacks [4]. There has been little research on the mental health of diplomatic personnel, with more attention paid to their physical protection than their psychological protection [4].

It is particularly relevant now to understand how to protect the mental health of diplomatic personnel, as during the COVID-19 pandemic diplomats have been required to continue carrying out their professional roles and balance their usual work pressures with the many risks, changes, challenges and uncertainties posed by the pandemic. The closing of borders has directly impacted diplomatic interactions and diplomats’ roles are likely to have changed, with staff required to monitor COVID-19 information and engage with the public in different ways [5]. Many will have been deployed to locations where healthcare (including mental healthcare) is limited and where they may not have their usual support networks. It has been suggested that international employees may have a particularly difficult time working effectively with different countries and cultures with the added uncertainty and unfamiliarity of working in a pandemic, and that the replacement of frequent social events common in international professions with stay-at-home restrictions could lead to a sense of loss [6] and difficulty adjusting to the host country [5]. Therefore, now is an important time—given that diplomatic organizations have a duty of care to protect the mental health of their staff—to further our understanding of diplomats’ well-being and how they might best be supported. Given the somewhat unique demands involved in diplomacy, it is useful to consider the mental health and well-being of diplomatic personnel as a distinct group. This scoping review was carried out with the aim of assessing what is already known about the mental health of those working in diplomatic roles.

## Methods

This review followed the Arksey and O’Malley’s scoping review framework [7]. The following question was identified: ‘What is known about the mental health and well-being of individuals working in diplomatic roles?’ This question was deliberately broad to encompass various aspects of well-being including the prevalence of mental health problems; factors potentially associated with well-being; and policies or interventions which may improve well-being.

In February 2021 the first author systematically searched four electronic databases (Embase, Medline, PsycInfo and Web of Science) using terms relating to diplomatic personnel (e.g. diplomats, ambassador, foreign office) and well-being-related terms (e.g. well-being, mental health, psychological). The full search strategy is available from the authors on request. Searches were updated in April 2022. The Hague Journal of Diplomacy was hand-searched, as were reference lists of included papers. Studies were eligible for inclusion if they: presented results of peer-reviewed research; were written in English; and included data on the mental health or psychological well-being of diplomatic staff or staff in very similar governmental roles. Case studies were excluded but any study with a sample size greater than one was relevant for inclusion. The first author carried out title screening, abstract screening and finally full-text screening to assess whether papers met the inclusion criteria. Any not meeting all criteria were excluded. Any uncertainties about inclusion were discussed with other authors.

Data (including design, participant characteristics, measures used and key results) were extracted onto a pre-designed spreadsheet. Principles from thematic analysis [8] were used to synthesize data. For example, any data relating to interventions trialled with diplomats were coded as ‘intervention’; similarities between codes were identified and codes were grouped into analytic themes (e.g. codes relating to the prevalence of stress or psychiatric conditions were included together within the ‘mental health problems or stress’ theme). Themes developed from the data were discussed with the other authors. Each of the themes identified in the data is summarized in the Results section below, with a narrative description of each theme and discussion of the evidence within each theme.

## Results

**Figure 1.**
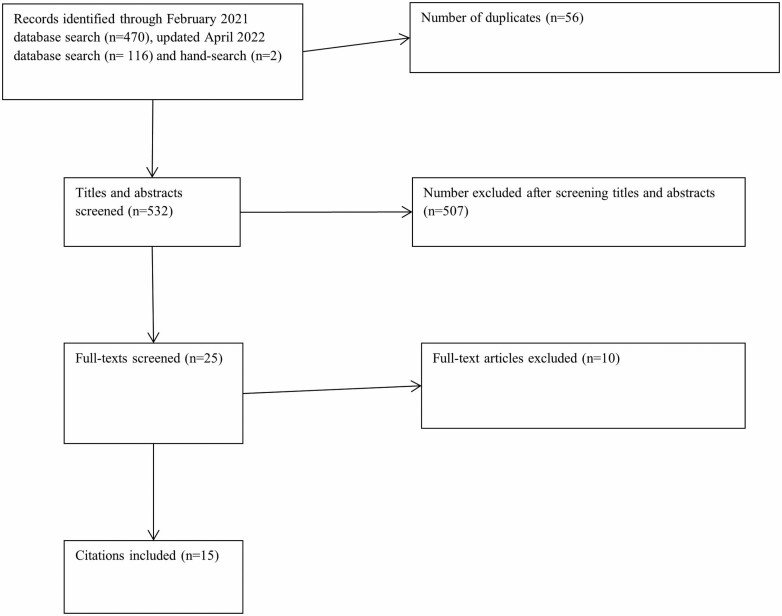
PRISMA flow diagram.

Data from 15 papers were reviewed and summarized here. Figure 1 illustrates the searching and screening process. Table 1 (available as Supplementary data at *Occupational Medicine* Online) summarizes the characteristics of the included studies.

The themes identified were: the psychological well-being of diplomats in terms of their quality of life or prevalence of mental health problems or stress [9–12]; factors associated with diplomats’ well-being [9,11,12]; diplomats’ perceptions of and satisfaction with their careers [13,14]; well-being of diplomats injured at work [15]; well-being specifically of trauma-exposed diplomats [16–21]; interventions for trauma-exposed diplomats [20–22]; and policies and practices for diplomats deployed to high-threat postings [23].

One German study [11] found that diplomats reported a significantly poorer quality of life than the general population, as well as significantly greater insomnia and fatigue. However, evidence from the UK suggested that diplomats reported low rates of psychiatric morbidity and fared better psychologically than other occupational groups whose work involved frequent international travel [12], although more poorly than their spouses. Evidence from the USA suggested diplomats who benefited from the US Department of State’s mental health service for diplomats fared better psychologically (in terms of post-traumatic stress disorder [PTSD] and suicide rates) than both military and civilian populations [10]. A study of diplomats in Iran [9] found that occupational stress was low overall, but significantly higher for staff who had musculoskeletal disorders; the same study found that burnout was higher in staff who smoked and staff with higher occupational stress. In a German study [11], individually perceived stress and job demands showed significant detrimental effects and personal resources (including self-efficacy, mobility-specific coping and social support) showed significant benefits to health-related quality of life (HRQOL). Female employees were more likely to report poorer HRQOL as were those posted abroad as opposed to residing in Germany; age, number of postings, occupational status (upper versus lower civil service grades) and time spent in international rotation did not appear to have an effect. Additionally, having children negatively affected HRQOL for women but not men.

Regarding career satisfaction, diplomats reported that repatriation frequently involved lateral or downward career moves, which was perceived as frustrating and difficult; there was some evidence the younger cohort were less willing to tolerate such career moves and more willing to leave if their aspirations for promotion were not met [13]. Retirees reported strong identification with, dedication to, and loyalty to the organization; younger ambassadors were less willing to tolerate the downsides of the job and were more likely to depart early [13]. Another study [14] found a negative relationship between career satisfaction and burnout, suggesting diplomatic expatriates who felt burnt out were less satisfied with their careers. Career satisfaction was not associated with gender, age, family situation or number of countries lived in, but those with high levels of identification with their home countries tended to have a more positive perception of their careers. Home country identification and host country identification collectively increased career satisfaction and moderated the relationship between burnout and satisfaction.

Green-McKenzie *et al.* [15] carried out a study with US diplomats injured during a work assignment in Cuba—diplomats had been exposed to high-pitched buzzing sounds and experiences of head/ear pressure, and subsequently experienced various cognitive, behavioural, emotional, vestibular, visual and auditory symptoms. None of their 45 participants met the criteria for severe anxiety or depression, although 11 reported minimal to moderate anxiety and most reported a low or average quality of life, with none reporting a high quality of life. There were no significant differences in psychiatric symptoms between those who had taken time off work and those who had not.

Six studies explored the psychological well-being of diplomatic personnel who had experienced potentially traumatic events, including war, terrorism and nuclear disaster [16–21]. Only one compared mental health of trauma-exposed diplomats to a non-trauma-exposed sample of diplomats, finding that those posted to war zones experienced significantly more PTSD symptoms but there were no significant differences between groups in terms of general psychiatric morbidity, fatigue or alcohol misuse [17].

Two studies explored post-traumatic reactions after a terrorist bombing in Kenya [18,19]. One-fifth (of *n* = 179) developed PTSD after the bombing [19]; US government employees had a significantly lower prevalence of bombing-related PTSD than civilians [19] or Red Cross staff [18] although this association did not remain significant in regression analysis [18]. Two studies reported on interviews with American diplomats, military and civilian personnel posted in Brussels having recently watched from afar the coverage of the September 11th attacks in the USA [20,21]. Many participants described feelings of dissociation, de-realization, numbness and detachment immediately following the incident, but as days passed they began to report hypervigilance, anxiety, poor concentration, anger, sleep difficulties, physical symptoms such as tightness in the chest and difficulty breathing, poor concentration and fears for the future [20,21]. Women had significantly higher stress responses, and participants aged 41 and over were significantly more affected by dissociative and arousal effects [21].

Government employees who had experienced the Fukushima nuclear meltdown also reported distress and PTSD symptoms and stressors such as unclear roles, high workload, handing over work to rapid deployment teams many of whom were not appropriately trained, office-based work, feeling that work was not immediately beneficial to the public, lack of breaks and difficulties with worried relatives back home [16]. Some reported a sense of ‘anti-climax’ after returning to usual duties, lack of time to adjust or receiving no proper follow-up afterwards which made them feel forgotten. However, participants also described their work positively, describing it as exciting and something they were proud of. Some believed they had learned more about themselves, been encouraged to take stock of their lives and felt a greater sense of camaraderie between colleagues.

Two studies by the same author [20,21] discussed stress debriefings with American diplomats posted in Brussels after the September 11th attacks in the USA. Debriefings involved a lecture describing and normalizing symptoms of acute distress and discussion of perceived current community threat levels. Participants reportedly found these sessions highly useful [20]; however, whilst discussing fears provided some relief to participants, it also potentially called group members’ attention to threats they had not considered, potentially causing ‘emotional contagion’ [21].

Greenberg *et al.* [22] explored the use of Trauma Risk Management (TRiM), a peer support intervention enabling personnel to manage the psychological needs of others who have been exposed to potentially traumatic events, in diplomatic personnel after the September 11th attacks. The TRiM Risk Assessment Tool found that distress symptoms became less common as time progressed after the event. Overall, TRiM was well-received by the diplomatic personnel and the Risk Assessment element of TRiM appeared to be able to measure changes in post-traumatic stress, leading the authors to suggest that TRiM might be a suitable alternative to traditional debriefing sessions.

Finally, Dunn *et al.* [23] surveyed personnel from international diplomatic organizations which routinely sent their staff to high-threat postings, finding little consensus on policies and practice for high-threat deployments. Most reported that specific interventions were provided for staff deployed to high-threat postings, including additional, individualized care and support (e.g. personal visits, emails, video meetings, mental health interventions); additional preparation (e.g. tailored briefings, specific training); enhanced leave; and additional physical and mental health assessments. Changes of tour length were typically allowed (by 86%), but there was inconsistent practice regarding altering tour lengths—50% carried out additional psychological evaluation as part of tour length alteration, 30% carried this out depending on the individual case and 20% did not carry out additional psychological evaluation.

## Discussion

This review is the first to collate existing literature on the mental health and well-being of staff working in diplomatic roles. Much of the data reported in the 15 included studies were disparate, with few studies investigating the same variables or outcomes; it is difficult to report much consensus in terms of what is known about diplomats’ well-being, and there are clear gaps in the literature.

Studies of well-being in the general diplomat population (i.e. not specifically trauma-exposed diplomats) yielded mixed results. None used standardized measures of common mental health disorders, instead measuring HRQOL, perceived stress or data showing the incidence of medical claims/psychiatric service use. Further research is needed to assess the prevalence of common mental health disorders in diplomatic personnel who are not deployed to hardship posts. Only three studies explored factors associated with diplomats’ mental health and as none examined the same predictors, it is difficult to draw any firm conclusions from these. We found some evidence that resources such as social support may be beneficial which is unsurprising, as social support has consistently been identified as protective of mental health and has been identified as a factor associated with positive adjustment and well-being in frequent business travellers [3]. Further investigation of factors affecting diplomats’ well-being is needed.

One study [18] suggested that identification with both home and host country was associated with better outcomes in terms of career satisfaction and lower levels of burnout, supporting suggestions that cross-cultural training could help to improve home and host country identification which could in turn protect diplomats’ mental health and well-being [24]. Another [13] noted differences in career perceptions between retired diplomats and a younger cohort of active diplomats, with the latter less likely to tolerate some of the negative aspects of the role and more likely to leave. This could be reflective of younger employees in general who may have different motivations and priorities to older cohorts [25]. In particular for diplomatic personnel, returning from abroad in lateral or downward moves (rather than promoting to high-ranked posts) appeared to be one of the factors affecting intentions to leave the organization, perhaps due to perceived loss of autonomy or loss of income [13]. It is therefore important for diplomatic organizations to ensure there are systematic policies in place regarding repatriation and promotion and to consider other ways of ensuring they retain their workforce.

The findings related to the mental health of trauma-exposed diplomats suggest that post-traumatic stress symptoms are common, especially immediately after the experience, and that post-traumatic growth can also occur; these trauma responses appear similar to those reported by other trauma-exposed occupational groups such as relief workers [26]. There was very little evidence relating to interventions for trauma-exposed diplomats; debriefings were self-reported to be helpful in two studies by the same author [20,21] but there were also concerns they could cause ‘emotional contagion’. It is notable that no well-accepted traumatic stress management guidelines support the use of such approaches and warn they may cause harm [27]. There was some evidence that TRiM could be helpful for trauma-exposed diplomats [22]; although further evidence is needed to ascertain its effectiveness specifically with diplomatic personnel, TRiM has been found to have a positive effect on organizational functioning among various other occupational groups [28].

Finally, based on the one study exploring the policies of diplomatic organizations [23], there do not appear to be standardized practices and policies in place across organizations to protect employees’ mental health when deployed to high-threat postings—despite diplomatic organizations having a duty of care to protect the mental health of their staff.

There are caveats to these findings: firstly, the relatively small and disparate data set. As only 15 articles were found, their results may not be generalizable to the wider population of diplomatic personnel, and since studies explored various different aspects of the diplomatic experience and looked at different outcomes and predictors, the evidence base for each finding is small. Additionally, we only searched four databases, and did not include grey literature: wider searches may have yielded additional data. Only one author carried out the searching and screening process: double-screening could have ensured that no relevant studies were excluded. Finally, as quality assessment of included papers is not typically required of scoping reviews, no formal appraisal was carried out although this could have strengthened the findings. Our observations were that several studies relied on cross-sectional data and opportunity sampling, and few considered confounding variables; intervention studies in particular tended not to be high-quality as they did not involve full trials to evaluate the interventions and instead relied on informal discussions with participants.

Our findings suggest that more research on diplomats not working in high-threat posts is warranted, due to the lack of research with this group. While diplomats in non-hardship posts may not be exposed to trauma, they are likely to be affected by similar non-traumatic stressors to those in high-threat posts, such as workload and high levels of responsibility. It was hoped that existing literature on diplomats’ well-being might help to understand how their psychological well-being could best be protected during a prolonged crisis such as COVID-19. However, given the lack of consistency and consensus in the research, it is difficult to use the current evidence base to develop guidelines for supporting diplomatic personnel. Until more research with this population is published, it is possible to draw on insights from other occupational groups which may be relevant to diplomatic organizations during times of crisis. For example, previous research on disaster-affected organizations highlights the impact that organizational climate can have, with recognition of staff performance, strong leadership and a supportive work culture associated with better well-being during disasters or crises [26]. Many of these factors are dependent on managers understanding and responding to the needs of their staff and so it is likely that ensuring those in supervisory/management positions have the skills and confidence to speak to team members about their well-being could be beneficial.

## Supplementary Material

kqad032_suppl_Supplementary_Table_S1
