# Supplementary material for: Mental health of diplomatic personnel: scoping review
Source: Occup Med (Lond). 2023 Mar 9;73(3):155–60. doi: 10.1093/occmed/kqad032 (PMC10132204; doi:10.1093/occmed/kqad032)
Supplement: kqad032_suppl_Supplementary_Table_S1 [file kqad032_suppl_supplementary_table_s1.docx]

*Table 1: Characteristics of literature on diplomats’ mental health and wellbeing*

| **Authors (year)** | **Country of authors** | **Focus of article** | **Participants** | **Participant demographics** | **Design** |
| --- | --- | --- | --- | --- | --- |
| Aghilinejad et al. (2014) [9] | Iran | Occupational stress and burnout in relation to musculoskeletal complaints in diplomatic employees | 161 employees of foreign embassies in Iran | 54% male  Mean age 39.3 | Cross-sectional quantitative survey assessing burnout, musculoskeletal complaints and workplace stress |
| Bakhshi et al. (2014) [16] | UK | Fukushima nuclear meltdown experiences of British Foreign Office staff in Japan | 36 British Foreign Office staff working in Japan | 61% male  Mean age 38 | Interviews exploring experiences of working during the disaster, perceptions of safety; interaction with British nationals; social support; coping; training; mental health; and perceptions of satisfaction/reward  Cross-sectional surveys assessing symptoms of distress and post-traumatic stress disorder |
| Dekleva (2017) [10] | USA | Overview of the US Department of State’s mental health service for US diplomats and their families | US diplomats and their families; the article does not report on participants of a study but instead reports on rates of use of psychiatric services | N/A | Comparison between rates of use of psychiatric services between diplomatic personnel/families and the general US population |
| Dunn et al. (2015) [23] | UK | International diplomatic organisations’ policies relating to high-threat postings | 14 staff from international governmental organisations that deploy diplomatic staff to high-threat postings (5 in human resources, 4 healthcare professionals, 4 occupational health practitioners / advisers and 1 policy-maker) | Gender not reported  Age not reported | Study-specific survey to assess organisational policies on deploying personnel to high-threat postings |
| Fliege et al. (2016) [11] | Germany | Health-related quality of life of diplomats | 2,433 German Foreign Service employees (70% posted abroad at the time of data collection) | 49% female  Mean age 44.9 | Cross-sectional quantitative survey assessing health-related quality of life, risk factors and protective factors |
| Green-McKenzie et al. (2022) [15] | USA | Clinical and psychological factors associated with return to work among US diplomats who experienced exposure to high-pitched loud noises, buzzing, and experiences of head/ear pressure whilst working in Havana, Cuba | 45 US diplomats referred to Occupational Medicine after the exposure | 60% male  Mean age 42.5 | Case series assessing medical and work history, physical health, work status, symptoms of mild traumatic brain injury, anxiety, depression, quality of life and sleep disturbance, at all Occupational Medicine visits between August 2017 – December 2019 |
| Greenberg et al. (2009) [22] | UK | Use of Trauma Risk Management (TRiM) for Foreign & Commonwealth Office staff In New York approximately two weeks after the September 11^th^ attacks | 20 Foreign & Commonwealth Office staff working in New York | Gender not reported  Age not reported | Follow-up quantitative study correlating TRiM risk assessment scores with scores on the Impact of Events Scale shortly after the event and again after one month |
| Hart & Baruch (2022) [13] | UK | Diplomats’ experiences of changes in the career structure of the civil service | 198 state ambassadors from the USA, UK, Israel and Denmark, including 119 recently retired diplomats and 79 active diplomats | 80% male  Mean age not reported; range 38-82 years | Qualitative study using semi-structured interviews to explore participants’ descriptions of and reflections on the changes that had occurred in their organisations |
| Hibberd & Greenberg (2011) [17] | UK | Mental and physical health of diplomats working in a war zone | 144 UK diplomats who had completed postings in Iraq or Afghanistan vs. 145 UK diplomats deployed overseas to non-hardship posts | Iraq/Afghanistan group: 60.7% male, mean age 41.9  Overseas group: 56.6% male, mean age 39.3 | Cross-sectional surveys assessing symptoms of common mental disorder; fatigue; post-traumatic stress disorder; alcohol consumption and harmful use; perceptions of the posting experience; exposure to trauma |
| North et al. (2018) [18] | USA | Post-traumatic stress disorder and other psychopathology after a terrorist bombing in Kenya | 99 locally engaged staff workers of the US government vs. 229 civilian employees and 64 workers of the Kenyan Red Cross Society | US government employees: 54% male, mean age 38.7  Entire study population: 52% male, mean age 34.6 | Diagnostic Interview Schedules assessing post-traumatic stress disorder; major depression; panic disorder; generalised anxiety disorder; alcohol use disorder |
| Patel et al. (2006) [12] | UK | Incidents of health events (i.e. incidences of illness or injury serious enough to require consultation with a doctor) in expatriates and factors associated with these events | 2,020 Foreign and Commonwealth staff and partners living abroad (1,287 staff and 733 partners of staff) | Staff: 69% male; mean age not reported but the most common age group was 30-39  Partners: 15% male, mean age not reported but the most common age group was 30-39 | 1-year cohort study examining the incidence of medical claims, health events, hospital admission and medical repatriation; Poisson regression to test for association between health events and various socio-demographic factors |
| Raitt et al. (2021) [19] | USA | Psychosocial effects following exposure to the 1998 terrorist attack on the US embassy in Nairobi, Kenya | 179 employees of the US government who were working in Nairobi at the time of the attack, including both Americans (n=53) and Kenyans (n=126) | 57% male  Mean age 40.6 | Cross-sectional survey carried out 8-10 months after the bombing, using a structured interview (to assess post-traumatic stress disorder, major depressive disorder, panic disorder, generalised anxiety disorder, somatisation disorder and alcohol use disorder), a companion interview (to collect information on disaster experiences, trauma exposure, injuries, subjective responses to the disaster and mental health treatment received) and a survey assessing safety concerns, worries, and coping strategies |
| Speckhard (2002) [20] | USA | Acute and posttraumatic stress responses in US military, foreign and civilian services serving overseas during the 9/11 terrorist attacks, and report on six stress debriefings carried out | 250 American diplomats, military and civilian personnel posted in Brussels | Gender not reported  Age not reported | Interviews assessing experience of acute stress disorder symptoms  Unstructured discussions with stress debriefing groups  Survey assessing acute stress disorder |
| Speckhard (2003) [21] | USA | Acute stress disorder in diplomats, military personnel and civilian Americans living abroad following the 9/11 terrorist attacks | 50 expatriate Americans living in Brussels at the time of the 9/11 attacks (62% diplomats or military personnel, 2% civilian non-government workers, 28% spouses, 8% who did not identify themselves) | 52% female, 42% male, 6% undisclosed  Mean age 42 | Surveys assessing dissociative symptoms; re-experiencing symptoms; avoidance; increased arousal; symptoms impairing psychological, social and occupational functioning; assault on world assumptions  Participants were asked to complete the surveys using recall of their responses the first week after the attacks and again at the time of the survey, 2-10 weeks after the attacks |
| Zhang et al. (2022) [14] | Finland, Denmark & Japan | Interplay between burnout, national identity and career satisfaction | 123 diplomats working outside their home country | 77% female  Mean age 43.9 | Cross-sectional surveys assessing career satisfaction; burnout; home and host country identification; and socio-demographic information |
